# Understanding Factors That Affect Willingness to Self-Manage a Pessary for Pelvic Organ Prolapse: A Questionnaire-Based Cross-Sectional Study of Pessary-Using Women in the UK

**DOI:** 10.1007/s00192-024-05840-1

**Published:** 2024-07-02

**Authors:** Lucy Dwyer, Azita Rajai, Dawn Dowding, Rohna Kearney

**Affiliations:** 1 Saint Mary’s Hospital, Manchester University NHS Foundation Trust, Manchester Academic Health Science Centre, Hathersage Road, Manchester, M13 9WL UK; 2 Division of Nursing, Midwifery and Social Work, School of Health Sciences, Faculty of Biology, Medicine and Health, University of Manchester, Oxford Road, Manchester, M13 9PL UK; 3 Manchester University NHS Foundation Trust, Manchester Academic Health Science Centre, Hathersage Road, Manchester, M13 9WL UK; 4 Centre for Biostatistics, Division of Population Health, University of Manchester, Manchester Academic Health Science Centre, Manchester, UK; 5 Institute of Human Development, Faculty of Medical & Human Sciences, University of Manchester, Oxford Road, Manchester, M13 9PL UK; 6 The Warrell Unit, Saint Mary’s Hospital, Manchester University NHS Foundation Trust, Hathersage Road, Manchester, M13 9WL UK

**Keywords:** Pessary, Prolapse, Self-management, Willingness, Genital self-image

## Abstract

**Introduction and Hypothesis:**

Pessary self-management offers benefits to women with no increased risk of complications. However, many are unwilling to self-manage, preferring clinician-led care. This study is aimed at exploring factors associated with willingness to self-manage a pessary.

**Methods:**

Women attending pessary clinic at a UK hospital were asked to complete a questionnaire providing responses on pessary use, comorbidities, female genital self-image, self-management experience and willingness (or not) to learn self-management. Based upon statistical advice we aimed to recruit 90 women. Data were analysed using the non-parametric Kruskal–Wallis test and Chi-squared test. Free text data were analysed thematically.

**Results:**

A total of 89 women completed the questionnaire. Thirty-three women (38%) had previously been taught pessary self-management. Of the remaining women, 12 (21%) were willing to learn, 28 (50%) were not willing and 16 (29%) were unsure. There was no correlation between female genital self-image and willingness to self-manage a pessary. Younger women were more willing to learn self-management (*p* =  < 0.001). Willing women were motivated by reduced follow-up visits. Self-managing women reported benefits including increased autonomy, cleanliness and giving their body “a break”. Reasons discouraging women from self-managing were a lack of confidence; feeling physically unable; wanting clinician-led care; fear of problems or previous problems with their pessary.

**Conclusions:**

Most women were either unsure about pessary self-management or unwilling to self-manage. Age was the only factor we found that had a significant relationship with willingness to self-manage a pessary. With robust self-management teaching, support and follow-up, it is likely that many of the barriers women report can be overcome.

## Introduction

Pessaries can effectively reduce symptoms of pelvic organ prolapse by providing structural support to the descending organs, thereby offering women an alternative to surgery [1]. Defining self-management is challenging owing to its complexity, use of differing terms such as self-care to describe self-management, and a lack of consensus regarding the requirements of a self-management intervention [2, 3]. However, for the purposes of this study, pessary self-management was defined as a woman independently removing and inserting her pessary. There is a lack of consensus regarding the necessary follow-up of self-managing women; however, it tends to be less frequent than women receiving clinician-based care [4]. In a randomised controlled trial of pessary self-management, self-managing women had a reduced risk of pessary complications whilst also attending less frequently for scheduled follow-up [5]. Furthermore, self-managing women report increased comfort, convenience, perceived access to help and support, and feeling of independence [4]. Despite these benefits, rates of willingness to self-manage a pessary varied between 3 and 83% of women in a recent scoping review of the literature [4]. The reasons for this significant variation in different studies is unclear. What is also unclear is what affects willingness to self-manage a pessary. Women have reported that age, experiencing pessary problems, a lack of confidence and perceived physical inability to self-manage a pessary are barriers for them [4]. However, this has not yet been rigorously measured in a population of pessary using women.

This study is aimed at exploring whether demographics, patient characteristics, previous pessary care or female genital self-image correlated with willingness to self-manage a pessary. Female genital self-image was included because a scoping review undertaken by the authors, identified that some women were unwilling to self-manage a pessary because they did not want to touch their genitals [4]. It has been established that women with low female genital self-image are less likely to perform a self-examination of their genitals as well as masturbating less frequently [6]. It was therefore hypothesised that there might a relationship between genital self-image and willingness to self-manage a pessary.

## Materials and Methods

In order to understand the complex issue of what affects willingness to self-manage a pessary, an explanatory sequential mixed methods approach was chosen. This enabled the researcher to analyse data from a large group of pessary-using women and explore the factors that correlate with willingness to self-manage a pessary for prolapse.

All women attending the pessary clinic at our institution who met the eligibility criteria were approached and given verbal and written information about the study. The inclusion criteria required women to be female adults who had retained a ring, shaatz, cube or inflatable pessary for at least 2 weeks, and to have both the capacity and ability to read and understand English in order to understand study participation and to complete the questionnaire. The types of pessaries specified in the inclusion criteria were based upon evidence that the majority of pessary practitioners in the UK do not feel that shelf and Gellhorn pessaries are suitable to be self-managed [7]. If women were willing to participate, they were asked to complete a short anonymous questionnaire. As approved by the Health Research Authority (22/SW/0102), written informed consent was not required, as completion of the anonymous questionnaire was deemed to imply consent. No payment or reimbursement was given for study participation owing to the low-impact nature of the study and because women did not incur any expenses as they were already present at the hospital for clinical care. Women who expressed a desire to complete the questionnaire at home were given a stamped, addressed envelope to minimise the burden and cost to them. Women were recruited between September and December 2022.

Data were collected via questionnaire, which requested information about the woman’s demographics, pessary use, self-management experience, willingness to self-manage and a free text box to express thoughts about pessary self-management. Women were also asked to complete the Female Genital Self-Image Scale (FGSIS-4), a reliable and validated four-item questionnaire that measures women’s attitude and beliefs about their genitals [8]. Scores in the FGSIS-4 range between 4 and 16, with a mean score of 12, in a nationally representative population of over 2,000 American women [6]. Herbenick et al. [6] have not determined a binary score for high and low FGSIS. However, for the purposes of this study, a score of eight or less was decided upon to indicate low FGSI, whereas a score of more than eight indicated high FGSI. The reasoning for this was, in order to score eight or less, a participant must disagree with all four statements describing positive genital self-image; therefore, this was deemed to accurately represent low genital self-image.

To determine a power calculation for our study, we hypothesised that female genital image was most likely to affect willingness to self-manage a pessary. Because the distribution between willingness to self-manage a pessary and FGSIS-4 has not yet been established, the sample size was calculated for a medium effect size of 0.3, for a Chi-squared test on a two by two table (self-management vs FGSIS). With 5% significance level a sample of 90 will have 80% power to detect a medium effect size of 0.3 [9]. Therefore, on this basis, we aimed to recruit 90 women to complete the survey.

Comparison between different levels of willingness and measures collected in the questionnaire was performed using a non-parametric Kruskal–Wallis test for continuous variables, and Chi-squared test for categorical variables. Effect of multiple testing was taken into account when interpreting the results and therefore p values close to 0.05 were not considered to be significant. Free text data were analysed thematically by two members of the research team, for the emergence of themes related to willingness, or not, to self-manage a pessary. While completing the FGSIS-4 questionnaire, eight women answered three of the four questions. In this instance, the unanswered question was replaced by the mean of the other three. Nine additional women had completed two or fewer of the FGSIS-4 questions and their data were excluded from FGSIS-4 analysis.

A pessary-using woman provided Patient and Public Involvement and Engagement (PPIE) for the study by confirming the importance of the research question to pessary-using women, reviewing the study protocol to confirm acceptability and providing feedback on the readability and accessibility of the study documents.

## Results

Ninety women completed the study questionnaire. However, the data of one woman were excluded after being recruited owing to the loss of mental capacity to consent and her questionnaire was securely destroyed. Therefore, a total sample size of 89 women were included in the analysis. Thirty-three women (38%) had previously been taught how to self-manage their pessary. Because this is not standard care at the organisation and optional for pessary-using women, this was interpreted as willingness to self-manage. Of the 56 women who had not previously been taught how to self-manage their pessary, only 21% (*n* = 12) were willing to attempt self-management (Table 1).

**Table 1:** Study population

	*N* = 89
Age median (IQR)	73.0 (62.0, 79.0)
Month using pessary, median (IQR)	48.0 (21.0, 84.0)
Know type of pessary (%)	54 (61.4)
Type of pessary (%)	
Cube pessary	2 (2.3)
Ring pessary	41 (46.6)
Ring pessary with support	5 (5.7)
Ring with knob	4 (4.5)
Shaatz pessary	2 (2.3)
Unsure	34 (38.6)
Know size of pessary (%)	18 (20.2)
Sexual orientation (%)	
Straight or heterosexual	85 (97.7)
Prefer not to say	2 (2.3)
Previously taught self-management (%)	33 (37.5)
Remove pessary independently (%)	31 (34.8)
Insert pessary independently (%)	29 (33.0)
How often remove or insert pessary?^a^ (%)	
A one-off event	6/32 (18.8)
Regularly remove and insert pessary	26/32 (81.25)
Willing to learn^b^ (%)	
Yes	12 /56 (21.4)
No	28/56 (50.0)
Unsure	16/56 (28.6)
Distance to hospital, miles, median (IQR)	5.90 (4.10, 8.80)
Deprivation, IMD, median (IQR)	5.0 (4.0, 7.0)
Education qualification (%)	55 (63.2)
Other qualification (%)	40 (62.5)
Degree level or above (%)	25 (52.1)
Ethnicity (%)	
White British	69 (77.5)
White Irish	12 (13.5)
Any other white background	4 (4.5)
Pakistani	3 (3.4)
Caribbean	1 (1.1)
Female Genital Self-Image Score	
1, median (IQR) appearance of genitals	3.0 (3.0, 4.0)
2, median (IQR) letting sexual partner look at genitals	3.0 (3.0, 3.0)
3, median (IQR) genitals smell	3.0 (3.0, 4.0)
4, median (IQR) embarrassment about genitals	3.0 (3.0, 4.0)
Total, median (IQR)	12.0 (12.0, 15.0)
Female Genital Self-Image Score = Low (%)	3 (3.8)
ACE-27 score, median (IQR)	0.50 (0.0, 1.0)
Number of comorbidities reported, median (IQR)	1 (0, 2) max = 5

*IQR* interquartile range, *IMD*, Index of Multiple Deprivation, *ACE-27* Adult Comorbidity Evaluation 27

^a^Percentages calculated from the 32 women who reported removing or inserting their pessary which was made up of women who had and hadn’t been taught pessary self-management

^b^Percentages calculated from the 56 women who had not been previously taught pessary self-management

The study was powered to determine whether there was a medium correlation between female genital self-image and willingness to self-manage a pessary. Our findings suggest that there might be no correlation between either mean FGSIS-4 and willingness to self-manage a pessary (*p* = 0.269), or low versus high FGSIS-4 score and willingness (*p* = 0.534). Statistical testing revealed that the only factor significantly correlated with willingness to self-manage a pessary was age (Table 2), with younger women being more willing to learn self-management. In women who had not previously been taught to self-manage a pessary, there was no significant relationship between willingness to learn self-management and whether or not women reported removing or inserting their pessary independently at home (Table 3).

**Table 2:** Descriptive statistics stratified by willingness to self-manage

*n* = 89	Willing to self-manage^a^, *n* = 45	Not willing to self-manage, *n* = 28	Unsure, *n* = 16	*p* value
Age, median (IQR)	65.00 (57.00, 73.00)	77.00 (74.75, 81.25)	73.00 (70.75, 78.25)	< 0.001
Month using pessary, median (IQR)	36.00 (22.50, 60.00)	66.00 (36.00, 120.00)	36.00 (7.50, 78.00)	0.053
Know type of pessary (%)	31 (70.5)	12 (42.9)	11 (68.8)	0.035
Size of pessary (mm), median (IQR)	72.50 (67.00, 77.00)	57.00 (56.50, 66.50)	70.00 (67.00, 70.50)	0.41
Know size of pessary (%)	12 (26.7)	3 (10.7)	3 (18.8)	0.28
Distance to hospital (miles), median (IQR)	5.90 (4.10, 9.90)	6.1 (3.60, 7.6)	6.20 (5.08, 6.50)	0.606
Deprivation, IMD, median (IQR)	5.00 (4.00, 7.00)	5.00 (3.00, 7.00)	6.00 (3.75, 6.25)	0.695
Certificated education qualification (%)	33 (75.0)	12 (42.9)	10 (66.7)	0.033
Ethnicity, mean (SD)				0.288
White British	33 (73.3)	23 (82.1)	13 (81.2)	Insufficient numbers to analyse
White Irish	5 (11.1)	4 (14.3)	3 (18.8)	
Any other white background	4 (8.9)	0 (0.0)	0 (0.0)	
Pakistani	3 (6.7)	0 (0.0)	0 (0.0)	
Caribbean	0 (0.0)	1 (3.6)	0 (0.0)	
Total FGSIS, mean (SD)	12.0 (11.0, 15.0)	12.0 (9.0, 13.0)	12.0 (9.8, 12.0)	0.1
FGSIS = low (%)	2 (4.8)	0 (0.0)	1 (6.2)	0.534
ACE-27 score, median (IQR)	0.00 (0.00, 1.00)	0.00 (0.00, 1.00)	1.00 (0.75, 1.25)	0.107
Number of comorbidities, median (IQR)	1.00 (0.00, 2.00)	1.00 (0.00, 2.00)	2.00 (2.00, 3.00)	0.044

*IQR* interquartile range, *IMD*, Index of Multiple Deprivation, *SD* standard deviation, *FGSIS* Female Genital Self-Image Score, *ACE-27* Adult Comorbidity Evaluation 27

For different levels of willingness, continuous variables were compared using non-parametric Kruskal–Wallis test and categorical variables were compared using Chi-squared test

*p* value from the tests are given

*p* value = NA where there was insufficient information

^a^Willing to learn self-management combined with women previously taught self-management

**Table 3:** Self-removal/insertion of pessary stratified by willingness to self-manage in women not previously taught pessary self-management

*n* = 55	Willing to self-manage, *n* = 12	Not willing to self-manage, *n* = 28	Unsure, *n* = 16	*p* value
Remove pessary independently (%)	4 (33.3)	3 (10.7)	1 (6.2)	0.103
Insert pessary independently (%)	3 (25.0)	2 (7.1)	1 (6.7)	0.221

Thematic analysis of the free text data revealed that women who were willing to learn self-management of their pessary, including those who had previously been taught, were motivated by the possibility of fewer hospital visits, with some recognising that this could have resource benefits for the NHS. *“Would allow appointment to be available to others with greater need. Would prefer a) to attend a local clinic and b) to do it myself”*. Women who were already self-managing their pessary reported the benefits of increased autonomy: *“In case I need to introduce or remove by myself”*; cleanliness: *“Self-managing is a great way to feel clean”*; and giving their body a break: *“To make it easier as sometimes I feel it's time to have a rest”*.

The barriers reported by women who were not willing to learn self-management of their pessary were a lack of confidence or feeling physically unable to self-manage their pessary: *“I'd be quite anxious I wouldn't insert it correctly and do damage but it could be ok.”*. Wanting to see a healthcare professional was cited: *“I feel a professional is best suited to do that. Whilst this is being carried out the womb can be checked at the same time”*. Some women perceived there to be a lack of robust follow-up for self-managing women: *“Not totally self-manage”*. Fear of problems with the pessary if self-managing or having prior experience with a pessary was also reported: *“Because size could be too painful in case is too big and we get regular check ups.”* and *“Have had so many problems—vaginal bleeding, allergic reaction to pessary, etc.* *that my check ups and vaginal examinations are essential to ensuring no problems that need attention”* and not perceiving self-management to be beneficial: *“I feel I don't want to and because my age I feel I don't need it”*. One woman also stated that she already felt too burdened by self-managing other conditions to undertake self-management of her pessary too: *“I already self catheterise and use Peristeen daily so I wouldn't want another responsibility”*.

## Discussion

This study was aimed at exploring whether factors including female genital self-image demographics, patient characteristics and previous pessary care affected willingness to self-manage a pessary. Our findings are that in women with no prior self-management experience less than a quarter were willing to learn how to self-manage their pessary. Furthermore, the only statistically significant factor that affected willingness to self-manage a pessary was age, with older women being less willing to self-manage a pessary for prolapse. Increasing age has been reported to be a barrier to pessary self-management anecdotally by participants in other studies [10, 11]. Furthermore, evidence suggests younger women are more likely to self-manage [12–18]. However, in a study of 779 women with a mean age of 64 years, 99% were able to perform self-care of their pessary [19]. The median age in this study was 73; therefore, the population was approximately 10 years older than in Koch et al.’s study [19], which is one explanation for the greater acceptance of self-management amongst these women. However, it may also be the case that despite older women being less willing to self-manage their pessary, most are able to, when it is accepted as standard care. Our findings also suggest that age affects willingness to self-manage a pessary, rather than simply access to the option of self-management, which is dependent upon the bias of pessary practitioners regarding who is willing to self-manage and who is capable of self-management. Furthermore, our findings suggest that age might be an independent factor that affects willingness to self-manage and that other confounding variables such as comorbidities do not appear to impact upon willingness. What is unclear is why willingness to self-manage a pessary would be affected by age. This warrants further exploration to determine whether additional support could be provided to older women to enable them to consider self-management thereby ensuring equitable access to this component of pessary care.

Our findings also revealed there was no difference between women who were willing or unwilling to self-manage their pessary and who already removed and inserted their pessary at home. This supports the findings from a study of elderly Canadian pessary-using women, who, despite having a high level of confidence in problem solving pessary problems by adjusting their pessary as required, declined self-management [10]. One potential reason for this hesitancy may be women’s perception of what pessary self-management is. As identified in the free text responses, a number of women in this study emphasised the importance of regular clinician-led follow-up to reassure them that everything was satisfactory. For some women, it was the reassurance of a health care professional examining them for both pessary-related complications and incidental findings. Other women felt reassured by a professional being responsible for pessary fitting. In Storey et al.’s study, the women reported that they viewed follow-up appointments as a social occasion and received valuable support from the pessary nurse that they would not get elsewhere [10]. Therefore, further research to explore pessary-using women’s preferences for care is necessary to enable us to understand what level of support and follow-up would reassure women that pessary self-management does not mean that they are discharged from a service and unable to access care and support in the future. Many of the barriers that women who were unwilling to learn self-management of their pessary reported, such as lacking confidence, concerns about ongoing support, fearing problems, and not understanding the benefits of self-management, could be addressed with a well-designed pessary self-management programme.

Several barriers reported by women in this study are similar to findings of other studies that have explored barriers and facilitators to self-management of chronic conditions. In a number of studies, women reported lacking confidence in their ability to self-manage [10, 20–24] and having concerns about or experiencing inadequate ongoing health care professional support [20, 21] were barriers to self-management. Furthermore, the literature suggests that addressing two barriers identified in this study—fearing problems and not understanding the benefits of self-management—facilitates self-management [23, 25]. Therefore, these barriers are not unique to pessary self-management and moreover, are reported specifically by women facing self-management of other chronic conditions. Well-designed pessary self-management support could address these barriers by empowering women to have confidence in their ability to self-manage and problem-solve, ensuring that women understand the benefits of pessary self-management, providing easy-to-access ongoing support, and reassuring women that self-management does not equate to being discharged from the pessary service.

Female genital self-image was not found to affect willingness to self-manage a pessary, despite our hypothesis that it might. It has previously been suggested that general body image does not impact upon willingness to self-manage a pessary [26]. However, because a barrier to pessary self-management reported by women in other studies was the intimate nature of touching one’s genitals [10, 11], and the Female Genital Self-Image Scale has been validated as a tool to measure self-examination and touching of the genitals [8], the FGSIS-4 was deemed to be more valid for this purpose. An unexpected finding of the study was that study participants had a median FGSIS-4 score of 12, which is equivalent to the median FGSIS-4 score in a nationally representative population of American women [8]. Owing to the nature of pelvic organ prolapse, it would not be surprising if women in this population had a lower genital self-image than the general population. As we only surveyed women who had established pessary management of their prolapse, it may be that pessary correction of their anatomy and resolution of their symptoms restores genital self-image to that of the general population. Alternatively, pelvic organ prolapse may not have an impact on women’s genital self-image. However, this has not previously been explored, and further research in this area would help clinicians to better understand the impact of prolapse on self-image.

Although the limitations of approaching a convenience sample were recognised, because such a large number of women attend the hospital pessary clinic each week, it was anticipated that various characteristics that may have an impact on willingness to self-manage a pessary would be represented within the sample. Manchester has a diverse population [27]; therefore, it was hoped that a representative sample of women would participate, despite being recruited from one site. The decision to only provide study literature in English was made for practical reasons. However, the requirement that women be able to speak and understand English, to give implied consent and complete the study questionnaire, will undoubtedly have affected the generalisability of our findings [28]. Every eligible woman who attended the pessary clinic during the recruitment period was approached and offered the opportunity to participate by questionnaire completion. Some women declined, but the number of women who did not want to participate and the reason for this were not recorded. This limits our understanding of the impact of selection bias upon the study findings, as potential reasons for non-participation include having particularly strong views against pessary self-management, not wanting to answer questions about female genital self-image or being disengaged with pessary care in general.

Owing to the vast majority of study participants being white, there was insufficient variation in the sample to analyse whether ethnicity affected willingness to self-manage. It has previously been established that white American women had higher levels of knowledge about pessaries for prolapse than American women from other ethnicities [29]. If white women are more aware of the possibility of treating prolapse with a pessary, they may be more likely to opt for pessary management. This may explain why such a high proportion of respondents to this questionnaire were white. However, it is not clear whether these racial disparities in pessary knowledge can be generalised to communities and countries. Therefore, further research is necessary to explore this issue in the UK population of equity of access to prolapse and pessary care regardless of ethnicity.

The decision was made to approach all women attending the pessary clinic to establish the proportion of pessary-using women willing to self-manage a pessary. However, it is acknowledged that because women who are already self-managing their pessary attend planned clinical follow-up less frequently at our organisation (annually instead of biannually), existing self-managing women would be sampled less frequently than women who were not currently self-managing. However, at the time of this study it was not our standard clinical care to routinely offer pessary self-management to women outside of recruitment to the Treatment of Prolapse with Self-Care Pessary study [2]. Therefore, only a small proportion of the population of pessary-using women at our hospital have previously been offered the option of self-management. Consequently, we believe that there is still a large number of pessary-using women attending the hospital who had not previously considered, or been aware of the possibility of, pessary self-management. However, we acknowledge the possibility of misclassification bias in our sample population.

The questionnaire was anonymous and self-completed by women in an attempt to minimise embarrassment while asking about personal questions such as female genital self-image, which may result in social desirability bias. However, this means that we are reliant upon the quality of responses provided by women, as we are unable to verify data using medical records, which would be particularly beneficial for the questions regarding co-morbidities. The Adult Comorbidity Evaluation 27 (ACE-27) questionnaire has been validated as a tool to calculate adult comorbidities, but it requires detailed information about current and historical medical conditions to determine the severity of comorbidities and calculate the subsequent score [30]. Without this level of information being provided by women, it is possible that the severity of conditions was underestimated when calculating the ACE-27 score, misrepresenting the health of the population. To mitigate this, as well as the ACE-27 score, we also analysed and provided details of the number of comorbidities that women reported. Although many of these conditions may seem minor, insignificant or historic to health care professionals and unlikely to have an impact on the ACE-27 score, the fact that the woman reported them indicates the significance to them and the burden that they feel in terms of self-managing their health and various conditions. As with the ACE-27 score, the number of conditions reported by women was low, indicating that overall, our population was in good health despite their age. It is possible that women with worse health or frailty felt less able to complete the study questionnaire.

## Conclusion

Three quarters of pessary-using women were unwilling or unsure about their willingness to self-manage their pessary. Female genital self-image did not affect willingness to perform pessary self-management. Other than age, no other characteristic was identified that has an impact upon willingness to self-manage. Participants reported a number of different reasons to explain unwillingness or uncertainty about willingness to self-manage a pessary and these findings should be incorporated into the design of robust pessary self-management programmes to ensure that women’s needs are met and that they are empowered to feel able to consider self-management and feel supported when doing so.

## Data Availability

All data relevant to the study are included in the article or available on request from the corresponding author.

## Declarations

### Regulatory approval

The study was approved by Bristol Research Ethics Committee and The Health Research Authority (Ref: 22/SW/0102).
